# Effects of Fluoride Varnish on *Streptococcus mutans* Count in Saliva

**DOI:** 10.5005/jp-journals-10005-1409

**Published:** 2017-02-27

**Authors:** Sourabh Badjatia, Rini G Badjatia, K Thanveer, Ajith CG Krishnan

**Affiliations:** 1 Senior Lecturer, Department of Public Health Dentistry, Modern Dental College & Research Centre, Indore, Madhya Pradesh, India; 2 Senior Lecturer, Department of Pedodontics and Preventive Dentistry, Modern Dental College & Research Centre, Indore, Madhya Pradesh India; 3 Professor, Department of Public Health Dentistry, K.M. Shah Dental College and Hospital, Vadodara, Gujarat; 4 Professor, Department of Public Health Dentistry, K.M. Shah Dental College and Hospital, Vadodara, Gujarat

**Keywords:** Dental caries, Fluoride varnish, *Streptococcus mutans.*

## Abstract

**Aim:**

To evaluate the effect of fluoride varnish on *Streptococcus mutans* count in saliva among 12-year-old school children.

**Materials and methods:**

A field experiment was conducted to evaluate the effects of fluoride varnish on S. *mutans* count in saliva among 12-year-old school children. A total of 42 school-going children attending schools in Vadodara district, Gujarat, India, were divided into two groups. Group I was treated with fluoride varnish and group II received no treatment. Assessment of S. *mutans* was carried out at baseline and 3 to 6 months postfluoride varnish application. Friedman analysis of variance test and *post hoc* test were applied to detect statistically significant differences between baseline, 3 to 6 months of fluoride varnish application, and also between groups I and II.

**Results:**

The mean number of salivary S. *mutans* value found in case group at baseline, 3 to 6 months was 31.23 ± 1.119, 9.27 ± 0.852, and 9.39 ± 0.908 × 10^4^ colony-forming unit CFU/mL respectively. The difference in S. *mutans* count from baseline to 3 to 6 months was highly statistically significant (p = 0.000), but the difference from 3 to 6 months was not statistically significant (p = 0.142). In control group, the mean *S. mutans* value found at baseline, 3 to 6 months was 30.63 ± 1.436, 31.23 ± 1.351, and 31.40 ± 1.374 × 10^4^ CFU/mL respectively. The differences between these values were not statistically significant (p = 0.11).

**Conclusion:**

Statistically significant reduction in S. *mutans* count in saliva was seen 3 to 6 months after fluoride varnish application.

**How to cite this article:**

Badjatia S, Badjatia RG, Thanveer K, Krishnan ACG. Effects of Fluoride Varnish on *Streptococcus mutans* Count in Saliva. Int J Clin Pediatr Dent 2017;10(1):62-66.

## INTRODUCTION

Dental caries is an ecological disease in which diet, host, and the microbial flora interact over a period of time in such a way as to encourage demineralization of the tooth enamel with resultant caries formation. Dental caries is still one of the most common diseases in the world.^1^

Dental caries will not occur if the oral cavity is free of bacteria. These bacteria are organized into a yellowish film known as dental plaque on the surface of the teeth. Many types of bacteria are present in the mouth; the most caries-active appear to be *Streptococcus mutans, Lactobacillus* spp., *Veillonella* spp., and *Actinomyces* spp. A variety of carbohydrates provide substrates for these organisms to grow on, and the waste products of their metabolism, acids, initiate the tooth decay process by dissolving tooth enamel.^1^

Research on the bacteriology of dental caries has focused on the ubiquitous *S. mutans* and its ability to ferment sucrose. It ferments sucrose to produce significant amounts of acid and extracellular polysaccharides (plaque).^1^ The most important understanding of caries process is that dental caries do not occur either in the absence of dental plaque or dietary fermentable carbohydrates. The *S. mutans* plays a significant role in the development of dental caries and is the chief pathogen.^2,3^

Oral colonization by *S. mutans* is required for dental caries initiation, and it has been suggested that an *S. mutans* count higher than 10^5^ colony-forming unit CFU/mL of saliva is related to higher caries risk. Hence, microbial monitoring has been considered as an alterna-five method for evaluating caries activity.^4^

As dental caries has multifactorial etiology, preventive measures usually involve a combination of dietary counseling, oral hygiene measures, and fluoride application.^5^ Fluoride-containing toothpastes (dentifrices), mouth rinses, gels, and varnishes are the modalities most commonly used at present, either alone or in combination.^6^ The use of fluoride varnish to prevent and control dental caries in children and adults is expanding in both public and private dental practice settings and in nondental settings that incorporate health risk assessments and counseling.^7^

Fluoride works primarily via topical mechanism inhibiting demineralization and enhancement of reminer-alization at the crystal surface and inhibition of bacterial enzymes.^8^ The most important anticaries effect of fluoride is considered to result from its action on the tooth/plaque interface, through promotion of remineralization of early caries lesions and by reducing tooth enamel solubility.^6^ Fluoride at low concentration is bacteriostatic and at high concentration, it is bacteriocidal.^7^ A high fluoride concentration in the oral cavity might inhibit acid production by bacteria and may reduce the number of certain species.^9^

Limited study, which explored the effect of fluoride varnish on *S. mutans,* does not show conclusive results. Moreover, the effect of fluoride varnish over a period of time has not been studied.

## AIM

To evaluate the effect of fluoride varnish on *S. mutans* count in saliva among 12-year-old schoolchildren.

## OBJECTIVES

- To estimate the count of *S. mutans* in saliva at baseline.
- To estimate the count of *S. mutans* in saliva 3 to 6 months after fluoride varnish application.
- To compare baseline salivary *S. mutans* count with that of 3 to 6 months postfluoride varnish application.

## MATERIALS AND METHODS

A field experiment was conducted to evaluate the effects of fluoride varnish on *S. mutans* count in saliva among 12-year-old schoolchildren of Vadodara district, Gujarat, India. The study protocol was reviewed and approved by the institutional ethics committee. Permission from respective authorities was taken prior to the study.

### Inclusion Criteria

Subjects having at least one carious tooth (clinical criteria)^10^ with salivary *S. mutans* count of ≥10^4^ CFU/mL of saliva (microbiological criteria).

### Exclusion Criteria

- Children who were caries free at baseline examination.
- Children whose parents refused to give informed consent.
- Children having history of antibiotics for past 3 to 4 weeks or were taking antibiotics.
- Children having history of fluoride treatment for past 6 months.

**Fig. 1:**
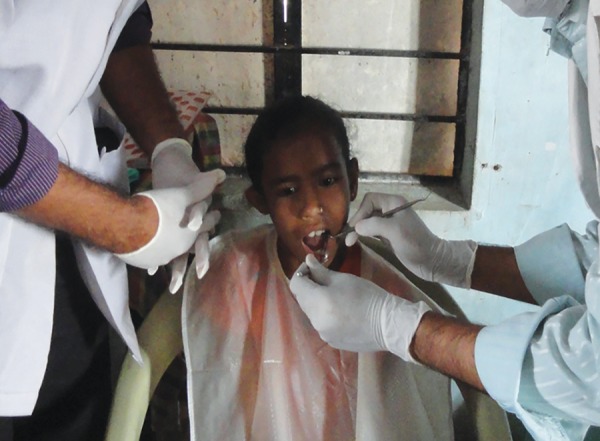
Examination of the study subjects for the presence or absence of dental caries

### Sample

All the 12-year-old schoolchildren were examined for the presence of caries (Fig. 1), out of which forty-two 12-year-old schoolchildren were selected based on inclusion and exclusion criteria.

The selected samples were then divided equally into two groups:

- *Group I:* Subjects who received fluoride varnish application at baseline (study group)
- *Group II:* Subjects who did not receive any fluoride varnish application (control group)

## METHODS

In the present study, data were collected by personal interview.

### Saliva Collection and Microbial Analysis

**Fig. 2:**
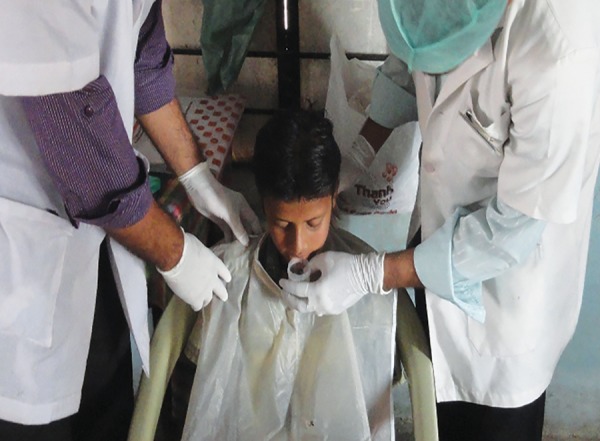
Collection of saliva of the study subjects for the purpose of microbiological evaluation

**Fig. 3:**
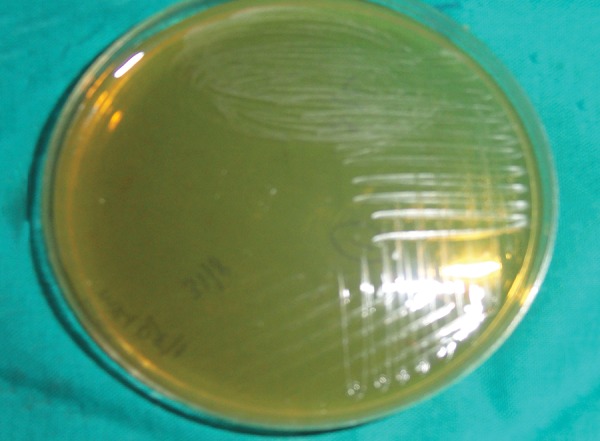
Colonies of S. *mutans* in the saliva of study subjects

Saliva collection and microbial analysis (Figs 2 and 3) was carried out three times for each study subject, i.e., before application of fluoride varnish and 3 and 6 months after fluoride varnish application. All salivary tests were carried out on whole saliva. About 2 mL of unstimulated saliva sample was collected. The participants were asked to refrain from eating and drinking anything for 1 hour before the collection. For the collection of saliva, the participants were seated comfortably, with their eyes open, and their head bent forward and asked to drool out saliva. The saliva was collected in a saliva-collecting chamber and sent on the same day for *S. mutans* analysis to the microbiology laboratory.

### Technique of Varnish Application

- *Step 1:* Teeth cleaned with the help of cotton and tweezers
- *Step 2:* Teeth dried with the help of chip blower.
- *Step 3:* A small amount of varnish (0.5 mL) will be dispensed
- *Step 4:* Varnish applied to the teeth with the help of applicator tip
- *Step 5:* Varnish was allowed to dry for 1 minute as per the instruction of manufacturer.

**Fig. 4:**
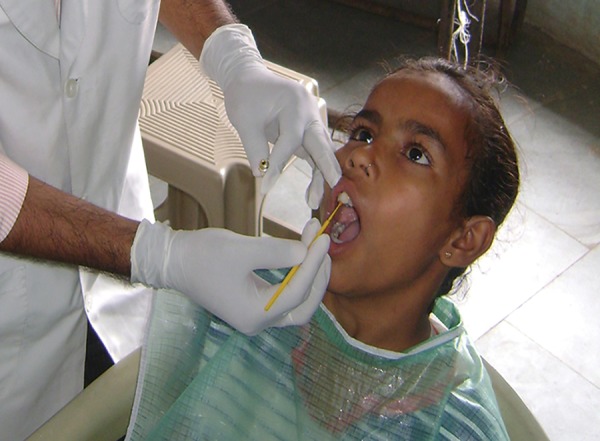
Application of fluoride varnish on the teeth of study subjects

These steps were performed quadrant-wise sequentially starting from the upper arch. Subjects were instructed not to rinse their mouth for 1 hour and not to eat for 4 hours (Fig. 4).

### Statistical Analysis

The data were analyzed using Statistical Package for the Social Sciences version 11. Friedman analysis of variance (ANOVA) test was used to compare *S. mutans* count at baseline, 3 to 6 months, and Mann-Whitney test was used to compare *S. mutans* count between group I (case group) and group II (control group).

## RESULTS

Data collected from the study subjects by personal interview reveals that all the subjects requited for the study had not undergone any antibiotic treatment in the past 6 months. Information regarding their oral hygiene practice, methods to clean their teeth, and their rinsing habits was also collected. Among the 42 subjects, 29 (69.0%) subjects cleaned their teeth once daily, 12 (28.6%) subjects cleaned their teeth twice daily, and 1 (2.4%) subject did not clean his/her teeth. About 24 (57.2%) subjects cleaned their teeth by using toothbrush, 10 (23.8%) used Datun, and 7 (16.6%) used finger. About 22 (52.4%) subjects cleaned their teeth by toothpaste, 12 (28.6%) by tooth powder, whereas 7 (16.6%) used other materials to clean their teeth. About 23 subjects (54.7%) rinsed their teeth once daily, 17 (40.5%) twice daily, and 2 (4.8%) after every meal. All the 42 (100%) subjects cleaned their teeth by using nonfluoridated toothpaste and had never received any fluoride treatment in the past 6 months.

Saliva sample analysis at baseline revealed that all the subjects who participated in the study had more than 10^4^ CFU/mL *S. mutans.*

Table 1 illustrates the number of *S. mutans* at baseline, 3 to 6 months after treatment with fluoride varnish in case group. The mean number of *S. mutans* value found at baseline, 3 to 6 months was 31.23 ± 1.119, 9.27 ± 0.852, and 9.39 ± 0.908 × 10^4^ CFU/mL respectively. The difference between these value was highly statistically significant; p = 0.00 (Friedman test, Chi square = 32.878).

**Table 1:** **Table 1:** Number of S. *mutans* at baseline, 3 to 6 months after treatment with fluoride varnish in case group

*Time*		*Subjects* * (n)*		*Mean ± SD* * (10^4^ CFU/mL)*		*Chi-square*		*p-val* *(Friedman* * ANOVA test)*	
Baseline		21		31.23 ± 1.119		32.878		0	
3 months		21		9.27 ± 0.852					
6 months		21		9.39 ± 0.908					

SD: Standard deviation

Table 2 shows *post hoc* comparison of *S. mutans* from baseline, 3 to 6 months after treatment with fluoride varnish in group I (case group). The difference in *S. mutans* count from baseline to 3 to 6 months was highly statistically significant (p = 0.000; Wilcoxon signed rank test), but the difference from 3 to 6 months was not statistically significant (p = 0.142; Wilcoxon signed rank test).

Table 3 projects the number of *S. mutans* from baseline, 3 to 6 months in group II (control group). The mean *S. mutans* value found at baseline, 3 to 6 months was 30.63 ± 1.436, 31.23 ± 1.351, and 31.40 ± 1.374 × 10^4^ CFU/mL respectively. The difference between these values was not statistically significant; p = 0.11 (Friedman test, Chi square = 6.000).

Table 4 illustrates *post hoc* comparison of *S. mutans* from baseline, 3 to 6 months after treatment with fluoride varnish in group II (control group). The difference in *S. mutans* count from baseline to 3 months, baseline to 6 months, and 3 to 6 months was not statistically significant (p = 1.000, p = 0.250 and p = 0.250 respectively; Wilcoxon signed rank test).

Table 5 shows comparison of *S. mutans* from baseline, 3 to 6 months between group I (case group) and group II (control group). The *S. mutans* value at baseline was not statistically significant (p = 0.194; Mann-Whitney Test), but highly statistically significant at 3 to 6 months (p = 0.000; Mann-Whitney Test).

**Table 2:** **Table 2:** *Post hoc* comparison of S. *mutans* at baseline, 3 to 6 months after treatment with fluoride varnish in case group

*Time*		*Subjects (n)*		*Wilcoxon signed rank test*	
Baseline to 3 months		21		Z = -4.016 p = 0.000	
Baseline to 6 months		21		Z = -4.025 p = 0.000	
3 to 6 months		21		Z = -1.527 p = 0.142	

**Table 3:** **Table 3:** Number of S. *mutans* at baseline, 3 to 6 months in control group

*Time*		*Subjects* * (n)*		*Mean ± SD* * (10^4^ CFU/mL)*		*Chi-square*		*p-val* *(Friedman* * ANOVA test)*	
Baseline		21		30.63 ± 1.436		6.000		0.11	
3 months		21		31.23 ± 1.351					
6 months		21		31.40 ± 1.374					

SD: Standard deviation

**Table 4:** **Table 4:** *Post hoc* comparison of S. *mutans* at baseline, 3 to 6 months after treatment with fluoride varnish in control group

*Time*		*Subjects (n)*		*Wilcoxon signed rank test*	
Baseline to 3 months		21		Z = -4.016 p = 1.000	
Baseline to 6 months		21		Z = -4.025 p = 0.250	
3 to 6 months		21		Z = -1.527 p = 0.250	

**Table 5:** **Table 5:** Comparison of S. *mutans* at baseline, 3 months, and after 6 months between case and control group

*Time*		*Subjects (n)*		*Mann-Whitney test*	
Baseline		42		Z = -1.314 p = 0.194	
3 months		42		Z = -5.572 p = 0.000	
6 months		42		Z = -5.567 p = 0.000	

## DISCUSSION

This is a unique study showing the effect of fluoride varnish on *S. mutans* count in saliva at baseline, 3 to 6 months.

Dental caries is a transmissible infectious disease in which *S. mutans* plays a major role. The *S. mutans* are generally considered to be the principal etiological agent of dental caries.

A measure of caries activity and caries risk is the concentration of cariogenic bacteria within saliva. Although *S. mutans* are most commonly associated with dental caries, several other microorganisms also have the ability to produce organic acids that induce demineralization of tooth structure and lead to clinically detectable caries.^11^

Caries activity is evaluated on the basis of data obtained from clinical examination and assessment of factors associated with the pathogenesis of the disease. These data can be collected by traditional visual inspection and probing or by some objective detection methods, which rely on the mineral changes as a basis for evaluation of caries activity and risk assessment. None of these methods aims at the estimation of the chief pathogen *S. mutans.* Now, microbial monitoring has been considered as an alternative method for evaluating current caries activity and future caries risk.^11^

The prevention of dental caries in children and adolescents is generally regarded as a priority for dental services and considered more cost-effective than its treatment.^6^ Fluoride therapy has been the centerpiece of caries-preventive strategies since the introduction of water fluoridation schemes over 5 decades.^12^

Fluoride varnish, which is also used as caries-preventive strategy, works by increasing the concentration of fluoride on outer surface of the teeth, thereby enhancing fluoride uptake during early stages of demineraliza-tion. Varnish hardens on the tooth as soon as it contacts saliva, allowing the high concentration of fluoride to be in contact with tooth enamel for an extended period of time (about 1-7 days). This is a much longer exposure as compared with other high doses of topical fluorides, such as gels or foams, which takes 10 to 15 minutes. The most important anticaries effect of fluoride results from its action on tooth and plaque interface, by promotion of remineralization of early caries lesions and reducing tooth enamel solubility.^13^

Hence, the present study was undertaken to compare the effect of fluoride varnish on *S. mutans* count in saliva at baseline, 3 to 6 months.

The subjects in the present study were accepted as a high-caries-risk group since they had at least one or more decayed tooth. The baseline salivary *S. mutans* levels were higher than 10^4^ CFU/mL.

The age of the present study population is around 12 years. This age is appropriate because it conforms to one of the World Health Organization indexed age groups.

In the present study, it was found that the baseline mean *S. mutans* count of group I was 31.23 ± 1.119 × 10^4^ CFU/mL. The mean value of *S. mutans* at 3 to 6 months after fluoride varnish application was 9.27 ± 0.852 and 9.39 ± 0.908 × 10^4^ CFU/mL respectively.

Whereas in group II, the mean *S. mutans* count at baseline, 3 to 6 months was 30.63 ± 1.436, 31.23 ± 1.351, and 31.40 ± 1.374 × 10^4^ CFU/mL respectively.

The difference in *S. mutans* count in group I from baseline to 3 to 6 months was highly statistically significant (p = 0.000; Wilcoxon signed rank test), but the difference from 3 to 6 months was not statistically significant (p = 0.142; Wilcoxon signed rank test) and also the difference in group II from baseline to 3 months, baseline to 6 months, and 3 to 6 months was not statistically significant (p = 1.000, p = 0.250, and p = 0.250 respectively; Wilcoxon signed rank test).

In the present study, the mean values of *S. mutans* from baseline, 3 to 6 months were compared between groups I and II. The *S. mutans* value at baseline was not statistically significant (p = 0.194; Mann-Whitney Test), but was highly statistically significant at 3 to 6 months (p = 0.000; Mann-Whitney test).

Zickert and Emilson^14^ found that the fluoride varnish treatment had no significant effect on the plaque and salivary levels of *S. mutans* at baseline and 4, 10, and 21 days after treatment with fluoride varnish.

Araujo et al^15^ observed that the application of fluoride varnish causes a significant suppression of *S. mutans* compared with baseline, 3 to 6 months.

Ekenbäck et al^16^ found no statistically significant difference for *S. mutans* in dental plaque after treatment with fluoride varnish at 1 week and 1 month relative to baseline.

### Recommendation

In the present study, children with at least one carious teeth and having *S. mutans* count at baseline of at least 10^4^ CFU/mL are included. Further studies with larger sample size and a placebo group can reveal the long-term effect of fluoride varnish on the *S. mutans* count in saliva of carious dentition.

## CONCLUSION

Fluoride varnish had a statistically significant reduction in the *S. mutans* count in saliva as compared with baseline, 3 to 6 months in our present study. Thus, we can conclude that fluoride varnish can be applied on teeth for broad spectrum antimicrobial activity, as an effective agent against *S. mutans* and in caries reduction.
